# Veterinary drug albendazole inhibits root colonization and symbiotic function of the arbuscular mycorrhizal fungus *Rhizophagus irregularis*

**DOI:** 10.1093/femsec/fiad048

**Published:** 2023-05-08

**Authors:** Eleni Gkimprixi, Stathis Lagos, Christina N Nikolaou, Dimitrios G Karpouzas, Daniela Tsikou

**Affiliations:** Department of Biochemistry and Biotechnology, University of Thessaly, Biopolis, 41500 Larissa, Greece; Department of Biochemistry and Biotechnology, University of Thessaly, Biopolis, 41500 Larissa, Greece; Department of Natural Resources and Agricultural Engineering, Agricultural University of Athens, 75 Iera Odos str., 11855 Athens, Greece; Department of Biochemistry and Biotechnology, University of Thessaly, Biopolis, 41500 Larissa, Greece; Department of Biochemistry and Biotechnology, University of Thessaly, Biopolis, 41500 Larissa, Greece

**Keywords:** albendazole, arbuscular mycorrhizal fungi, plants, symbiosis, toxic effects, veterinary drugs

## Abstract

Arbuscular mycorrhizal fungi (AMF) are plant symbionts that have a pivotal role in maintaining soil fertility and nutrient cycling. However, these microsymbionts may be exposed to organic pollutants like pesticides or veterinary drugs known to occur in agricultural soils. Anthelminthics are veterinary drugs that reach soils through the application of contaminated manures in agricultural settings. Their presence might threaten the function of AMF, considered as sensitive indicators of the toxicity of agrochemicals to the soil microbiota. We determined the impact of the anthelminthic compounds albendazole and ivermectin on the establishment and functionality of the symbiosis between the model-legume *Lotus japonicus* and the AMF *Rhizophagus irregularis*. Our analyses revealed negative effects of albendazole on the development and functionality of arbuscules, the symbiotic organelle of AMF, at a concentration of 0.75 μg g^−1^. The impairment of the symbiotic function was verified by the reduced expression of genes *SbtM1, PT4* and *AMT2;2* involved in arbuscules formation, P and N uptake, and the lower phosphorus shoot content detected in the albendazole-treated plants. Our results provide first evidence for the toxicity of albendazole on the colonization capacity and function of *R. irregularis* at concentrations that may occur in agricultural soils systematically amended with drug-containing manures.

## Teaser

Veterinary drug albendazole negatively affects the function of the symbiotic relationship between plants and arbuscular mycorrhizal fungi.

## Introduction

Soil microorganisms are pivotal in ecosystem functioning; they are involved in nutrient cycling (Fierer 2017), modulate soil structure (Rillig et al. 2015), degrade and detoxify pollutants (Fenner et al. 2013) and eventually engage into symbiotic relationships with plants enhancing plant growth and tolerance to biotic and abiotic stressors (Trivedi et al. 2020). However, their health and functioning could be compromised by chemicals, like pesticides and veterinary drugs that, intentionally or unintentionally respectively, reach soil. The potential toxicity of pesticides on soil microorganisms has attracted much attention (Feld et al. 2015, Vasileiadis et al. 2018, Romdhane et al. 2019, Zhang et al. 2019) and certain tests, like the Organisation for Economic Co-operation and Development (OECD) N transformation tests, are used at risk assessment level (OECD 2000). However, several publications have highlighted the need for advancements in the assessment of the toxicity of pesticides on soil microorganisms, suggesting the introduction of novel and standardized molecular tools (Martin-Laurent et al. 2013, Karpouzas et al. 2022). In this frame, the European Food Safety Authority (EFSA) Panel on Plant Protection Products and their Residues (PPR), recommended adding tests with arbuscular mycorrhizal fungi (AMF) to the data requirements and risk assessment, besides retaining and advancing the N-transformation test (EFSA Panel on Plant Protection Products and their Residues 2017).

AMF are mutualistic symbionts that associate with most land plants and are considered a key group in soil systems. They provide important ecosystem services by influencing the terrestrial C cycling (Parihar et al. 2020) and improving the soil fertility (Fall et al. 2022). The plant-AMF association is ancient and one of the best studied symbiotic relationships in nature. AM symbiosis provides the plant with mineral elements (mainly phosphorus and nitrogen), improves water absorption and enhances the plant tolerance to biotic and abiotic stresses (Smith and Read 2008, Begum et al. 2019). The symbiotic organelle of the AM symbiosis, the arbuscule, is a highly branched tree-like structure that forms within cells of the inner root cortex and facilitates the nutrient exchange between the plant cell and the fungus (Paszkowski 2006). Both phosphate and ammonium transporters have been identified to function in the arbuscule-containing cells and mediate the transfer of phosphorus and nitrogen from the fungus to the plant, like the phosphate transporter PT4 (Volpe et al. 2016) and the ammonium transporter AMT2;2 (Guether et al. 2009) in the model legume *Lotus japonicus*.

The toxicity of pesticides on AMF has been the subject of several *in vitro* (Buysens et al. 2015) and soil studies (Jin et al. 2013, Karpouzas et al. 2014), with effects ranging from negative to positive or neutral (Hage-Ahmed et al. 2019). The potential inclusion of AMF into the list of EFSA for Ecological Risk Assessment biological indicators, arises the need for the optimization of the relevant testing protocols (Sweeney et al. 2022). The current standardized method (reviewed and confirmed in 2020) uses an *in vitro* spore germination bioassay of the mycorrhizal fungus *Glomus mosseae* (ISO/TS 10832 : 2009). However, Mallmann et al. (2018) recommended a modification of the specific ISO test with the inclusion of other AMF species and test conditions relevant to the climatic zone where the pesticide is going to be authorized. Although spore germination is an important trait of the pre-symbiotic phase, other parameters should also be considered since effects may occur at both asymbiotic and symbiotic stages. The breadth of available methodologies to test pesticides against AMF, ranging from *in vitro* bioassays to field trials, is reviewed in Sweeney et al. (2022).

Beside pesticides, a number of ecotoxicological studies have explored the effects of pharmaceuticals, mostly of antibiotics, on soil microorganisms (Ding and He 2010). For example, it was shown that tetracycline exhibited chronic inhibitory effects on the growth of nitrifying bacteria (Katipoglu-Yazan et al. 2015), while the regular application of tylosin, sulfamethazine and chlorotetracycline in soil induced changes in the diversity of nitrogen fixing bacteria in soybean roots (Revellin et al. 2018). Such findings forced researchers to call on an increased consideration of microbial endpoints in antibiotics environmental risk assessment (Brandt et al. 2015), which can be further extended to other pharmaceuticals reaching agricultural soils like veterinary drugs. Unlike veterinary antibiotics, the potential effects of anthelminthic veterinary drugs on soil microorganisms, including keystone functional groups like AMF, remain unknown. It is now well-documented that anthelminthics, used to control infestations of productive animals by gastrointestinal nematodes (Kaplan 2020), are not metabolized in animal body and they are excreted in animal feces (Aksit et al. 2015). These are then used as manures in agricultural soils contributing to the dispersal of anthelmintic compounds in agricultural soils (Iglesias et al. 2018, Porto et al. 2021). Upon their transfer in agricultural soils, anthelminthics like benzimidazoles (albendazole) and macrocyclic lactones (ivermectin and eprinomectin) would interact with the soil biota and/or transfer to surface or groundwater systems (Sim et al. 2013) or taken up by plants (Mesa et al. 2020) imposing a threat for the environment and human health (Navrátilová et al. 2021). Most of the studies to date have focused on the potential effects of anthelmintics on soil fauna (Verdú et al. 2018, Barrón-Bravo et al. 2020), while little, if anything, is known about their off-target effects on the soil microbiota.

In the current study, we tested the potential toxicity of two of the most widely used anthelmintic compounds globally, albendazole and ivermectin, on the establishment and functioning of the plant-AMF symbiosis. For our tests we used the model legume *Lotus japonicus* and the commercially available AM fungus *Rhizophagus irregularis* strain DAOM. Different concentrations of the tested compounds were applied to our system and the development and functionality of the symbiotic relationship were monitored by microscopic, physiological and molecular analyses. Our results provide unprecedented evidence for the toxic potential of the anthelmintic compound albendazole to the establishment and functioning of AMF-plant symbiosis.

## Materials and methods

### Biological material, inoculation, and growth conditions

The plant used in our assays was the *Lotus japonicus* L. ecotype Gifu B-129 wild-type. It was inoculated with *R. irregularis* strain DAOM (Agronutrition). *Lotus japonicus* seeds were surface scarified, sterilized and kept in water for 12 h at 4°C, then germinated for 10 days at 21°C (16 h light, 8 h dark). Plant seedlings were transferred to magenta boxes containing 360 g baked sand and treated with 60 ml Long-Ashton nutrient solution (SLA) (Hewitt 1952). Each magenta box contained three plants and 300 spores of the AMF inoculum (almost 100 spores per plant) inside single sandwiches composed of nitrocellulose discs (Giovannetti et al. 1993) (Fig. S1). Plants were grown at 24–25°C (16 h light, 8 h dark) and harvested at four- or five-week post inoculation in experiment 2 and 1, respectively.

### Application of anthelminthics

Analytical standards of the anthelminthic compounds ivermectin (97% purity, Sigma-Aldrich, St Gallen Switzerland) and albendazole (98% purity, Tokyo Chemical Industry, Zwijndrecht, Belgium) were used in our study. The main physicochemical and environmental fate properties of the anthelminthic compounds tested are presented in Table S1. Dense solutions of both compounds in DMSO (10 mg ml^−1^) were initially prepared. These were further diluted in Long-Ashton nutrient solution (SLA) to prepare working solutions of each compound in the intended concentration range. The working solution was then applied as a single application at the start of the experiment to the baked sand in the magenta boxes. Control treatments received SLA amended with DMSO but without any anthelminthic compounds. In all cases, the amount of DMSO in the working solutions was 0.1%. All experiments were performed in five biological replicates for each treatment.

### Experiment 1–effects of veterinary drugs on AMF root colonization and P content of plants

We first tested the toxic effects of albendazole and ivermectin on the colonization of the plant root by AMF. *L. japonicus* plants were inoculated with the fungus *R. irregularis* and treated with different concentrations of either albendazole (0.5, 5, and 15 mg L^−1^) or ivermectin (0.5, 5, and 50 mg L^−1^), while a set of samples served as controls (see above). These concentrations are within the levels reported in soils (Thiele-Bruhn 2003, Liebig et al. 2010, Babić and Mutavdžić Pavlović 2013, Belew et al. 2021) and they correspond to exposure scenarios expected to occur in agricultural soils amended rarely (0.5 mg L^−1^ corresponding to nominal concentration in soil of 0.083 mg kg^−1^) or regularly (5 mg L^−1^ corresponding to nominal concentration in soil of 0.83 mg kg^−1^) with contaminated manure or used as dumping sites for contaminated manure (15 or 50 mg L^−1^ corresponding to nominal concentration in soil of 2.5 or 8.3 mg kg^−1^, respectively). The percentage of mycorrhizal root colonization was determined five weeks later, as well as the phosphorus content of albendazole-treated plants, as described below.

### Experiment 2- effects of veterinary drugs on the functioning of AMF

Based on the outcome of experiment 1, a second experiment was employed to verify via molecular means the inhibitory effect of albendazole on AMF functioning and shed light on the potential mechanisms of its toxicity on AMF. Similarly to the first experiment, *L. japonicus* plants were inoculated with the fungus *R. irregularis* and treated with different concentrations of albendazole (0.5 and 5 mg L^−1^). A set of samples were treated with SLA without any albendazole to serve as control. All plants were harvested at 4 weeks and the expression levels of three AM symbiosis marker genes was determined by RT-q-PCR. The transcript levels of *SbtM1* (Takeda et al. 2009) are indicators of normal stage transition during arbuscule development, while the transcript levels of *PT4* (Volpe et al. 2016) and *AMT2;2* (Guether et al. 2009) are used as indicators of the phosphate and ammonium import activity from the fungus to the plant, respectively.

### Determination of the levels of albendazole in the sand

Albendazole was extracted from 5 g of magenta sand with 10 ml of acetonitrile through shaking for 1 h on a horizontal orbital shaker at 220 r/m. The samples were then centrifuged for 5 min at 7500 r/m, and the supernatant was collected. The sand was re-extracted with a further 10 ml of acetonitrile. The supernatant from the two extraction steps were combined and passed through 0.45-μm hydrophobic syringe filters (PTFE Syringe Filter) before HPLC analysis. The concentration of albendazole in the sand was determined by HPLC analysis in samples taken at zero, two and four weeks after application.

HPLC analysis was performed in a Shimatzu HPLC-DAD system equipped with a Grace 297 Smart RP C18 (150 mm × 4.6 mm). Albendazole was detected at 205 nm using a mobile phase of acetonitrile/ phosphoric acid solution (0.1%) at a 20/80 v/v ratio. The flow rate of the mobile phase was 1 ml min^−1^. More details about HPLC conditions and the validation of the extraction method can be found in Lagos et al. (2022).

### Quantification of mycorrhizal colonization

AMF-colonized roots were stained with 5% ink in 5% acetic acid solution (Vierheilig et al. 1998) and the levels of colonization were quantified by microscopic examination of slides. Each slide was prepared from a sub-sample of the roots of the three plants grown per magenta box and contained almost 20 cm of root. The (%) percentage of arbuscule formation was estimated by the examination of at least 100 eyepieces per slide. Five slides were examined per treatment, corresponding to five biological replicates.

### Phosphorus plant content measurement

Fifteen to twenty mg dried above-ground plant material from *L. japonicus* plants were heated to ash at 550 °C, and then the ash was solubilized with 1 ml of 67% HNO_3_. The shoot extract was diluted to six ml with distilled water. Total concentration of P was determined with a PG T60 UV/ VIS Spectrophotometer, at 880 nm wavelength, following the Murphy and Riley color reaction method.

### RNA extraction and RT-q-PCR analysis of AM symbiosis marker genes

Total RNA was extracted from plant roots by a modified Lithium Chloride-TRIzol LS (Thermo Fisher) protocol (Holt et al. 2015). RNA concentration was determined using a microvolume spectrophotometer (DNA/Protein Analyse) (Quawell). RNA was DNAse treated using DNAse I (Thermo Scientific) according to manufacturer guidelines. cDNA was prepared using 300 ng of total RNA, an oligo dT primer and SuperScript II (Invitrogen) according to manufacturer guidelines. RT-q-PCRs reactions were performed using KAPA SYBR® FAST qPCR master mix (KAPABIOSYSTEMS) and 200 nM primer concentration. Levels of target genes were normalized to levels of two reference genes: *L. japonicus ATP SYNTHASE2* (*LjATP2*) and *L. japonicus PROTEIN PHOSPHATASE2a* (*LjPP2a*). RT-q-PCR reactions were executed in a BioRad CFX Connect lightcycler (BioRad). The LinRegPCR software was used for the data analysis (Ruijter et al. 2009). All primers are listed in Table S2.

### Statistical analysis

**Figure 1.**
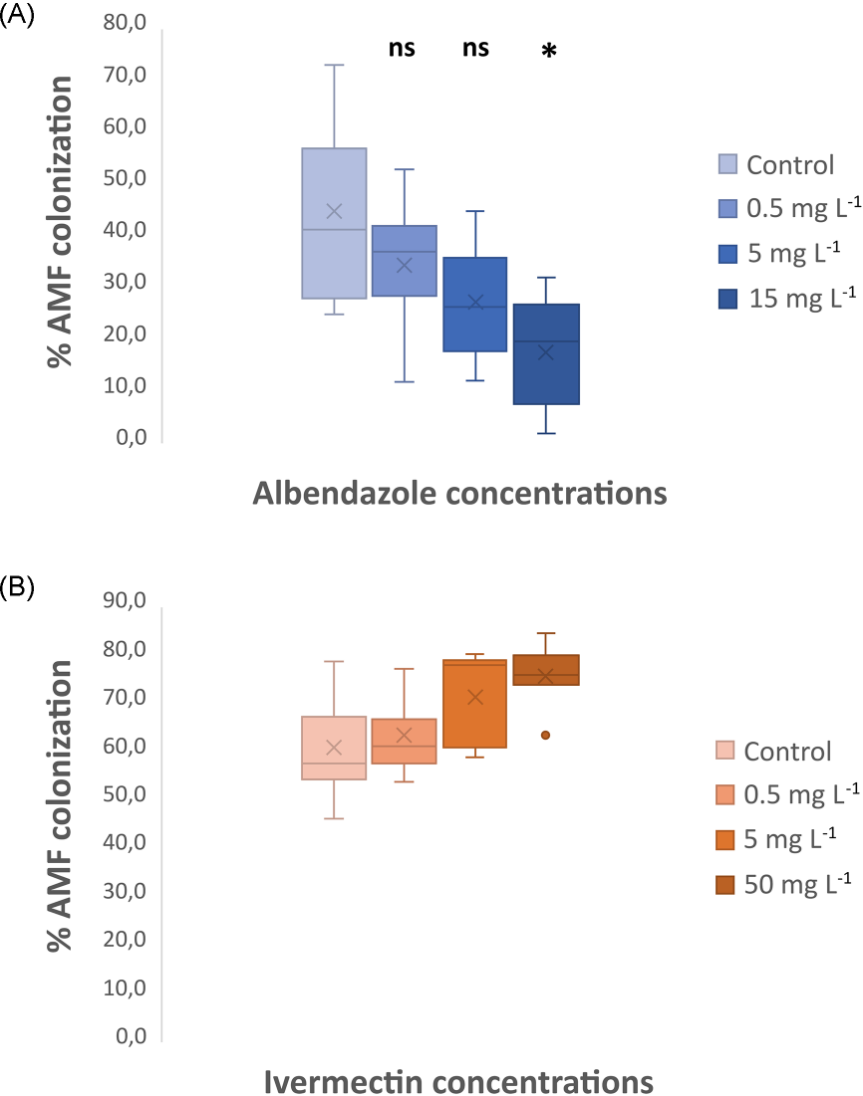
Arbuscular mycorrhizal fungi (AMF) root colonization levels after application of veterinary drugs. **(A)** Application of albendazole affects AMF root colonization. **(B)** Application of ivermectin does not affect AMF root colonization. Levels of AMF root colonization five weeks after the application of different concentrations of albendazole or ivermectin (or DMSO for the control treatment). Comparisons are between drug-treated and DMSO-treated (control) plants of *L. japonicus*. Statistical analysis was performed by *t-*tests: *P<0.05 (ns = not significant). No statistically significant difference was detected for invermectin treatments. (n = 5)

**Figure 2.**
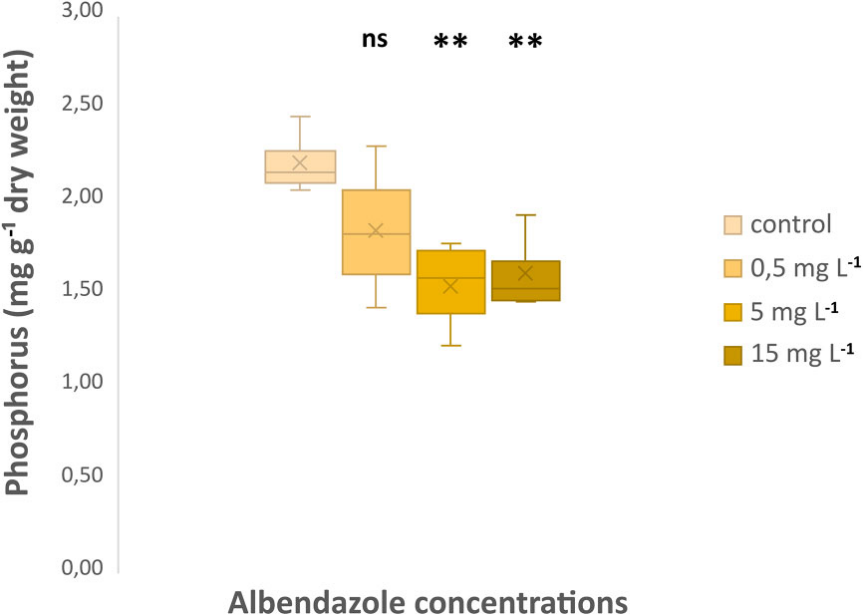
Application of albendazole decreases phosphorus content in the plant shoot. Phosphorus content in the shoots of *L. japonicus* plants five weeks after the application of different concentrations of albenzole (or DMSO for the control treatment). Comparisons are between albendazole-treated and control plants. Statistical analysis was performed by *t*-tests: **P<0.01 (n = 4) (ns = not significant)

**Figure 3.**
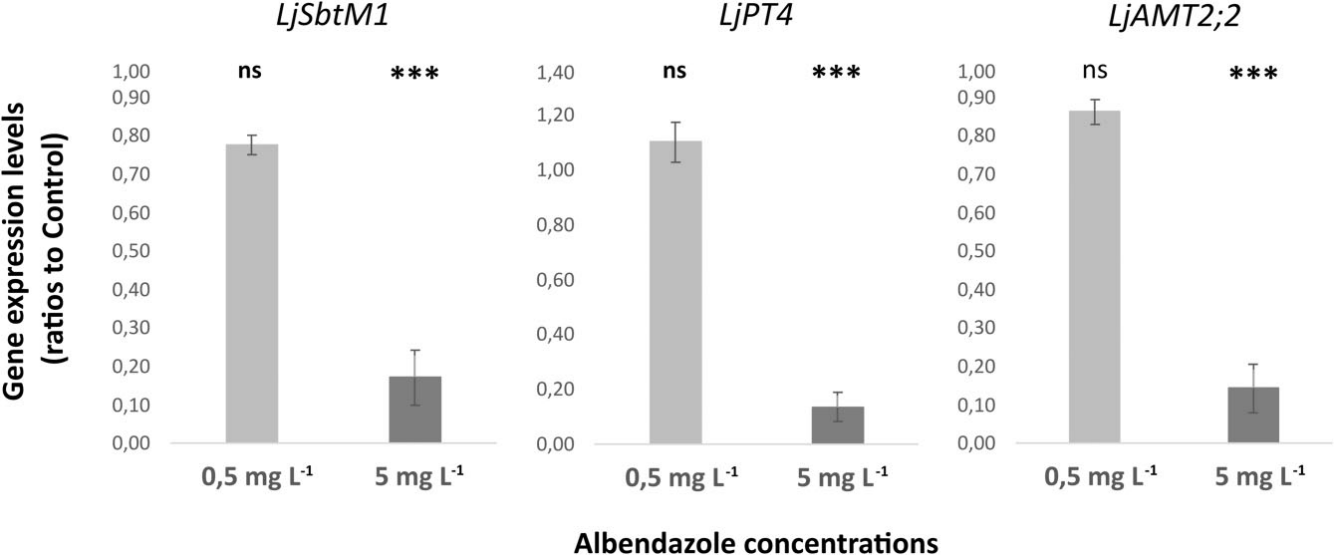
Application of albendazole affects the expression levels of mycorrhizal marker genes. Ratios of gene expression levels in the roots of albendazole-treated to DMSO-treated (control) *L. japonicus* plants. Bars show means ± SE (n = 5). Comparisons are between albendazole-treated and control plants. Statistical analysis was performed by *t*-tests: ***P<0.001 (ns = not significant)

To identify the drug concentration that may affect the establishment and functionality of the symbiotic relationship, pairwise comparisons between drug-treated and drug-untreated (control) root samples were employed (Figs 1, 2, and 3). Statistical analyses were performed by Student's *t-tests*. A significance level of 5% was applied.

To test if there is a dose effect of albendazole on AMF root colonization, the Pearson's correlation coefficient was measured (between albendazole concentration and % AMF root colonization). The correlation was found significant at the level of 0.05 and the value of the correlation coefficient r was −0.532 (negative correlation, *P* value of 0.019) (Fig. S2).

**Figure 4.**
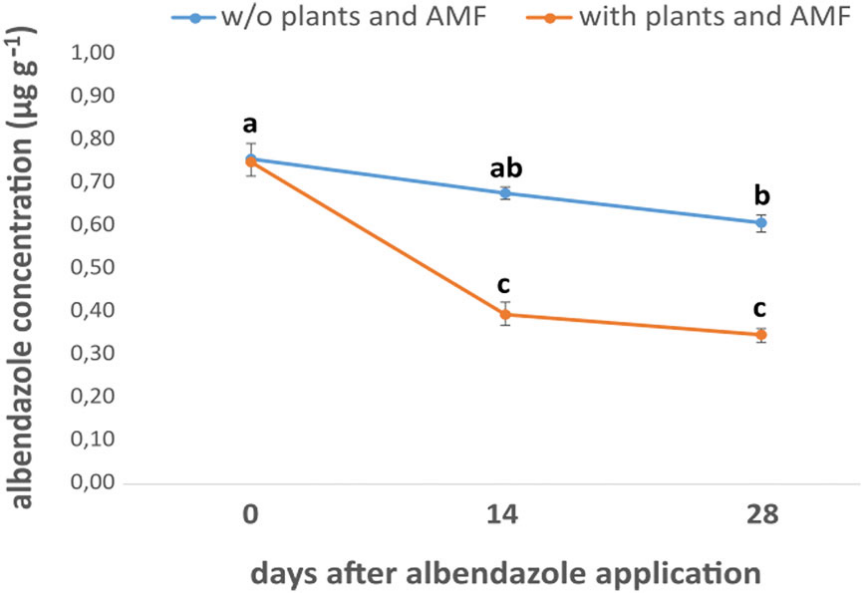
Levels of albendazole in the plant growth substrate. Albendazole concentration was determined by HPLC analysis in sand samples from magentas boxes with or without plants and AMF. The initial concentration of albendazole in the solution was 5 mg L^−1^. Statistical analysis was performed by one-way ANOVA followed by Tukey's post-hoc test (three technical replicates) (*P<0,05*). Significant differences are indicated by different letters.

Considering the albendazole dissipation experiment (Fig. 4), in order to compare the albendazole concentration between different treatments and time points, statistical analysis was performed by one-way ANOVA followed by a post-hoc Tukey's test.

## Results and discussion

### Albendazole negatively affects AM root colonization and P uptake by plants

Application of albendazole solution with a concentration of 15 mg L^−1^, corresponding to a measured concentration in sand of 2.49 μg g^−1^, resulted in significantly reduced levels of mycorrhizal colonization compared to the control treatment (Fig. 1A, Fig. S2). No significant differences were observed when solutions with lower albendazole concentrations (0.5 and 5 mg L^−1^ corresponding to measured concentrations of 0.055 and 0.765 μg g^−1^ in sand, respectively) were applied to our system (Fig. 1A), however, a negative dose effect of albendazole was detected on the AMF root colonization by measuring the Pearson's correlation coefficient (Fig. S2). Contrastingly, application of ivermectin in the plant substrate did not have any effect on the mycorrhizal root colonization, even when a high concentration of 50 mg L^−1^ was tested (Fig. 1B). The differential activity of albendazole and ivermectin against AMF root colonization may be attributed to the different mode of action of the two compounds. Ivermectin may not impede AMF root colonization as it has been reported to act as an allosteric modulator of glutamate-gated chloride channels in nematodes and insects, and also, ion channels of the host central nervous system (Martin et al. 2021), a mode of action not relevant for AMF and/or their plant host. On the other hand, benzimidazoles, like albendazole, act by inhibiting the polymerization of β-tubulin during microtubule formation in mitosis (Ramírez et al. 2001), a fundamental biological mechanism in the microbial world and beyond. Although this is the first report regarding the toxicity of albendazole to off-target microorganisms like AMF, previous studies have highlighted the inhibitory effects of benzimidazoles like the fungicides benomyl and carbendazim on AMF. These compounds are strong inhibitors of AM fungal colonization (Sukarno et al. 1993, O'Connor et al. 2009) and P uptake (Larsen et al. 1996, Kling and Jakobsen 1997, Schweiger and Jakobsen 1998) with effects observed at agricultural relevant dose rates.

To find out whether albendazole has a direct effect on the plant-AMF symbiosis or the reduced mycorrhizal colonization phenotype results from a general effect of this compound to the plant development, we monitored the plant growth by measuring the shoot and root length. No differences were detected in either shoot or root length of the plants treated with increasing concentrations of albendazole (Table 1). This observation is in line with previous findings, as albendazole did not affect the germination of mustard seeds (Prchal et al. 2016).

**Table 1.** Application of albendazole does not affect plant growth.

	Shoot length (cm)	Root length (cm)
control	4,3 ± 0,2	5,1 ± 0,2
0,5 mg L^−1^	4,2 ± 0,2	5,2 ± 0,3
5 mg L^−1^	4,1 ± 0,2	5,5 ± 0,2
15 mg L^−1^	4,2 ± 0,1	5,4 ± 0,2

Measurements were performed five weeks after the application of different concentrations of albendazole (or DMSO for the control treatment).

No significant difference was detected between treatments (n = 5).

To further explore if the observed inhibitory effect of albendazole on AMF colonization translates into an impairment of phosphorus (P) uptake by plants, we monitored the P levels in the plant shoots. We noted that the plants treated with solutions of 5 or 15 mg L^−1^ albendazole (corresponding to 0.765 and 2.49 μg g^−1^, respectively in sand) accumulate significantly lower levels of P in the shoot tissues, compared to control plants not receiving albendazole (Fig. 2). No significant changes were detected in the shoot P content when plant roots were treated with solutions of 0.5 mg L^−1^ of albendazole (corresponding to 0.055 μg g^−1^ in sand) (Fig. 2).

Overall, our results indicate that albendazole interferes with processes underlying the establishment of the symbiotic relationship and impairs the capacity of plants for P uptake but has no effect on the plant host.

### Albendazole affects arbuscule development and functionality

Following up on the observation that albendazole reduces the P content of plant shoots, we determined, in a second experiment, the expression levels of three genes known to be involved in arbuscules development, and arbuscule-mediated P and N uptake by plants. The expression levels of all three marker genes were found significantly reduced in the roots of plants treated with a solution containing 5 mg L^−1^ of albendazole (corresponding to 0.754 μg g^−1^ measured concentration of albendazole in sand), compared to the control plants (Fig. 3). The levels of *LjSbtM1* were almost 6-fold decreased, and the levels of *LjPT4* and *LjAMT2;2* were almost 7-fold decreased. This dramatic reduction in the transcripts of the AM marker genes denotes impaired development and functionality of the arbuscules formed on these roots. Treatment of plants with solutions of a lower concentration of albendazole, 0.5 mg L^−1^ (corresponding to 0.055 μg g^−1^ in sand) did not result in significant changes in the gene transcript levels, compared to the control plants (Fig. 3).

Overall, the results obtained from the molecular analysis confirmed our observations from the microscopic analysis; albendazole has a direct negative effect on AM symbiosis. Although the microscopic analysis did not detect impairment of the AMF root colonization when plants were treated with a solution of 5 mg L^−1^ of albendazole (Fig. 1), this was detected by the RT-q-PCR analysis and concurred with the significant reduction in P uptake by plants (Fig. 2) when exposed to the same concentration levels of albendazole. Based on these results we suggest that an adequate number of arbuscules are formed on the roots of the plants treated with the solution of 5 mg L^−1^ of albendazole, however, these arbuscules are not well developed and fully functional. Our results indicate that the presence of albendazole in the soil at concentration levels that are expected to be found in regularly manured agricultural soils has toxic effects on the symbiosis of the fungus *R. irregularis* with the model legume *L. japonicus*, resulting in limitation of the fungus potential to provide the plants with essential nutrients like phosphorus. More in depth analysis *via* transcriptomic approaches are needed to establish the full extent of the response of AMF to albendazole exposure.

### Albendazole dissipation

We also followed the dissipation of albendazole in our system to verify the level and the duration of the exposure of the plant-AMF system to the anthelminthic compound. Albendazole showed a slow dissipation in the absence of plant and AMF with more than 80% of its initially detected amount remaining in the system at the end of the study (Fig. 4). In contrast, in the presence of plants and AMF we noted a significant dissipation of albendazole which exceeded 50% of its initial concentration level. These results suggest that the presence of plants and AMF facilitates the removal of albendazole from sand. In the absence of an established biota in our sand system, beyond AMF and the roots of *L. japonicus*, we speculate that the gradual reduction of albendazole levels is driven either by an increasing uptake of the anthelminthic compound by plant roots or by a transformation of albendazole by AMF or by a combination of both activities. The capacity of plants to uptake albendazole through their root system has been previously shown (Stuchlíková Raisová et al. 2017), while the contribution of AMF in the enhancement in the degradation of organic pollutants like pesticides has been also reported (Wang et al. 2011). However, it should be stressed that the dissipation patterns of albendazole in our system are expected to differ from its environmental fate in agricultural soils. The dissipation of anthelmintics, including albendazole, in soil is driven by biotic (microbial degradation) and abiotic processes (adsorption and leaching), which are less important in our system, and their relative contribution to anthelminthics dissipation is affected by soil properties like pH and organic carbon content (Mutavdžić Pavlović et al. 2018, Porto et al. 2021, Lagos et al. 2022).

## Conclusions

We provide first evidence for the toxicity of the antlhelminthic benzimidazole compound albendazole, which unlike the macrocyclic lactone ivermectin, inhibited the plant colonization capacity and function of the AMF *R. irregularis* with the latter constituting a more sensitive and ecotoxicologically relevant endpoint to follow. Further tests will expand our toxicity testing to the full range of anthelminthic compounds with the aim to benchmark the current assay as a tool for assessing the potential toxicity of veterinary drugs like anthelminthics to the soil microbiota. This study provides the first indication that veterinary drugs like anthelminthics may harm beneficial plant symbiotic interactions and highlight the need for consideration of key soil microbial groups like AMF in ecotoxicological testing of pharmaceuticals, that may reach the soils through the use of manure and sludge as organic fertilizer, or through irrigation of agricultural fields with treated wastewater. The results of such tests are expected to contribute to the design of ecofriendly and safe agricultural practices in the future.

## Supplementary Material

fiad048_Supplemental_File
