# Supplementary material for: Veterinary drug albendazole inhibits root colonization and symbiotic function of the arbuscular mycorrhizal fungus Rhizophagus irregularis
Source: FEMS Microbiol Ecol. 2023 May 8;99(6):fiad048. doi: 10.1093/femsec/fiad048 (PMC10696295; doi:10.1093/femsec/fiad048)
Supplement: fiad048_Supplemental_File [file fiad048_supplemental_file.docx]

**Supplementary Data**

**Veterinary drug albendazole inhibits root colonization and symbiotic function of the arbuscular mycorrhizal fungus *Rhizophagus irregularis***


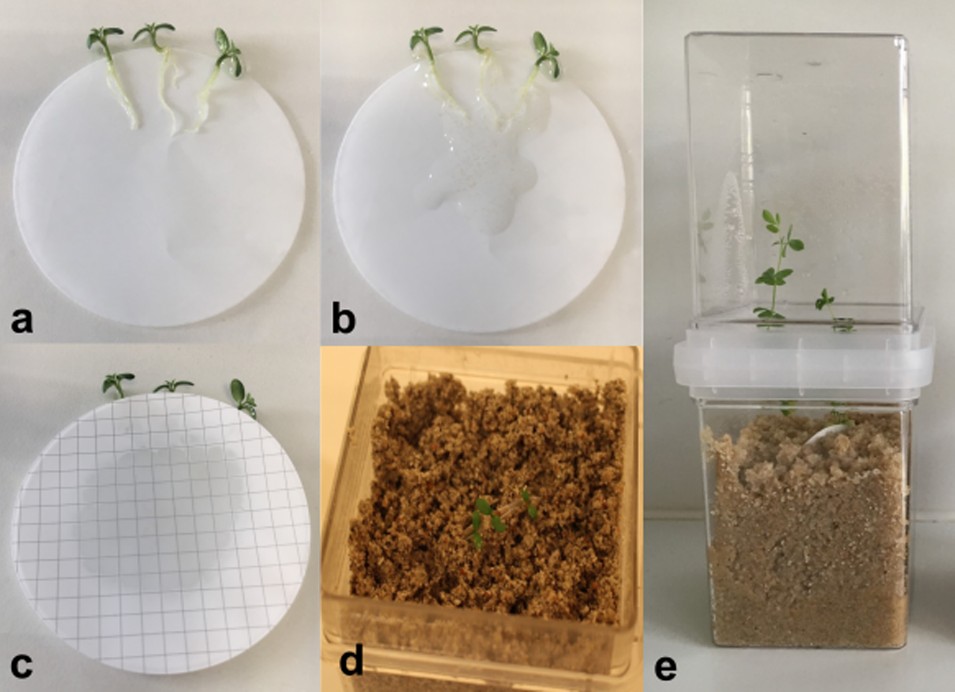


**Fig S1. The “sandwich” method:** **a**) three plants are placed on a pre-wet nitrocellulose filter, **b**) *R. irregularis* inoculum is applied at a concentration of 100 spores per plant, **c**) a second nitrocellulose filter covers the roots and the inoculum, **d**) the sandwich is placed in a magenta box filled with baked sand and medium, **e**) plants grow in double magenta boxes. (The method was described in Giovannetti et al. 1993).

**Fig S2. AMF root colonization is negatively correlated to albandazole.** The Pearson correlation between albendazole concentration and AMF root colonization was found significant at the level of 0.05 and the value of the correlation coefficient r was -0,532 (negative correlation, P value of 0.019), denoting a dose effect of albendazole on the AMF root colonization.

**Table S1**. The chemical structures and main physicochemical properties of the anthelmintic (AH) compounds studied

| Compounds | Chemical structure | Molecular weight (g mol^-1^) | Water solubility (mg L^-1^) | Log K_ow_ | pKa | DT_50_ in soil  (days) | Koc  (L kg^-1^) |
| --- | --- | --- | --- | --- | --- | --- | --- |
| Albendazole | 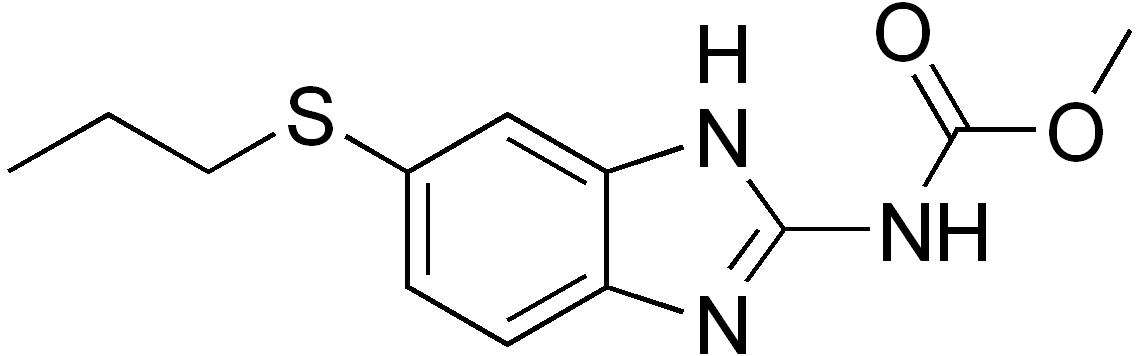 | 265.3 | 41.0 **^a^** | 3.14 ^b^ | 3.37, 9.93 ^c^ | 0.1-4.2^d^ | 1553.4-3561.3^e^ |
| Ivermectin | 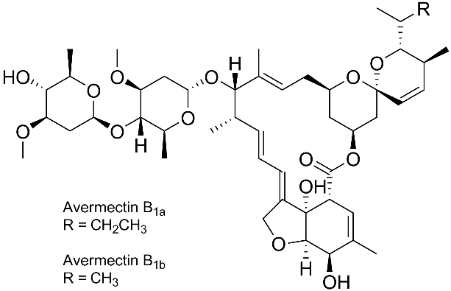 | 875.1 | 4.0 ^f^ | 5.6 ^g^ | No pKa between pH 3-10 | 15.7-66.1^d^ | 4000-25800^h^ |

^a^ PubChem [Internet]. Bethesda (MD): National Library of Medicine (US), National Center for Biotechnology Information; 2004-. PubChem Compound Summary for CID 2082, Albendazole; [cited 2022 Mar. 21].

^b^ Tomasz G, Jan JJ, Walerian P. Correlations between no observed effect level and selected parameters of the chemical structure for veterinary drugs. *Toxicology in Vitro* 2010;**24**:953–959

^c^ Takacs-Novak K, Jozan M, Szasz G. Lipophilicity of amphoteric molecules expressed by the true partition coefﬁcient. *International Journal of Pharmacology* 1995;**113**:47–55

^d^ Lagos S, Moutzoureli C, Spiropoulou I *et al.* Biodegradation of anthelmintics in soils: does prior exposure of soils to anthelmintics accelerate their dissipation. *Environmental Science Pollution Research* 2022;**29**:62404–22

^e^ Pavlovic DM, Glavac A, Gluhak M, Runje M. Sorption of albendazole in sediments and soils: Isotherms and kinetics. *Chemosphere* 2018;**193**:635-644

^f^ Fent GM. *Encyclopedia of Toxicology (Third Edition)*, Academic Press, 2014

^g^ Heinrich AP, Zoltzer T, Bohm L *et al.* Sorption of selected antiparasitics in soils and sediments. *Environmental Science Europe* 2021;**33**:77

^h^ Krogh, KA, Soeborg T, Brodin B, Halling-Sorensen B. Sorption and mobility of ivermectin in different soils. *Journal of Environmental Quality* 2008;**37**:2202–2211

**Table S2:** List of primers used in the RT-q-PCR analysis

| **Gene** | **Forward Primer** | **Reverse Primer** | **Reference** |
| --- | --- | --- | --- |
| *LjATP2* | CAATGTCGCCAAGGCCCATGGTG | AACACCACTCTCGATCATTTCTCTG | Tsikou et al., 2018 ^1^ |
| *LjPP2a* | GTAAATGCGTCTAAAGATAGGGTCC | ACTAGACTGTAGTGCTTGAGAGGC | Tsikou et al., 2018 |
| *LjSbtM1* | CAGGTGAACCAGAAGGTTGCATAC | AGCAGCACCCTCTCTATCTTCATGC | Rasmussen et al., 2016 ^2^ |
| *LjPT4* | CCAGAACCTCACACAGAAAGACATC | AACACGGTGAACCAGTACCCTGG | Rasmussen et al., 2016 |
| *LjAMT2;2* | ACACATGCTTGCACTGCTACC | CTGCCCATCCTTGAACAACCC | Guether et al., 2009 ^3^ |

^1^ Tsikou D, Yan Z, Holt DB *et al.* Systemic control of legume susceptibility to rhizobial infection by a mobile microRNA. *Science* 2018;**362**:233-236.

^2^ Rasmussen SR, Füchtbauer W, Novero M *et al.* Intraradical colonization by arbuscular mycorrhizal fungi triggers induction of a lipochitooligosaccharide receptor. *Sci Rep* 2016;**6**:29733.

^3^ Guether M, Neuhäuser B, Balestrini R *et al.* A mycorrhizal-specific ammonium transporter from *Lotus japonicus* acquires nitrogen released by arbuscular mycorrhizal fungi. *Plant Physiology* 2009;**150**:73-83.
